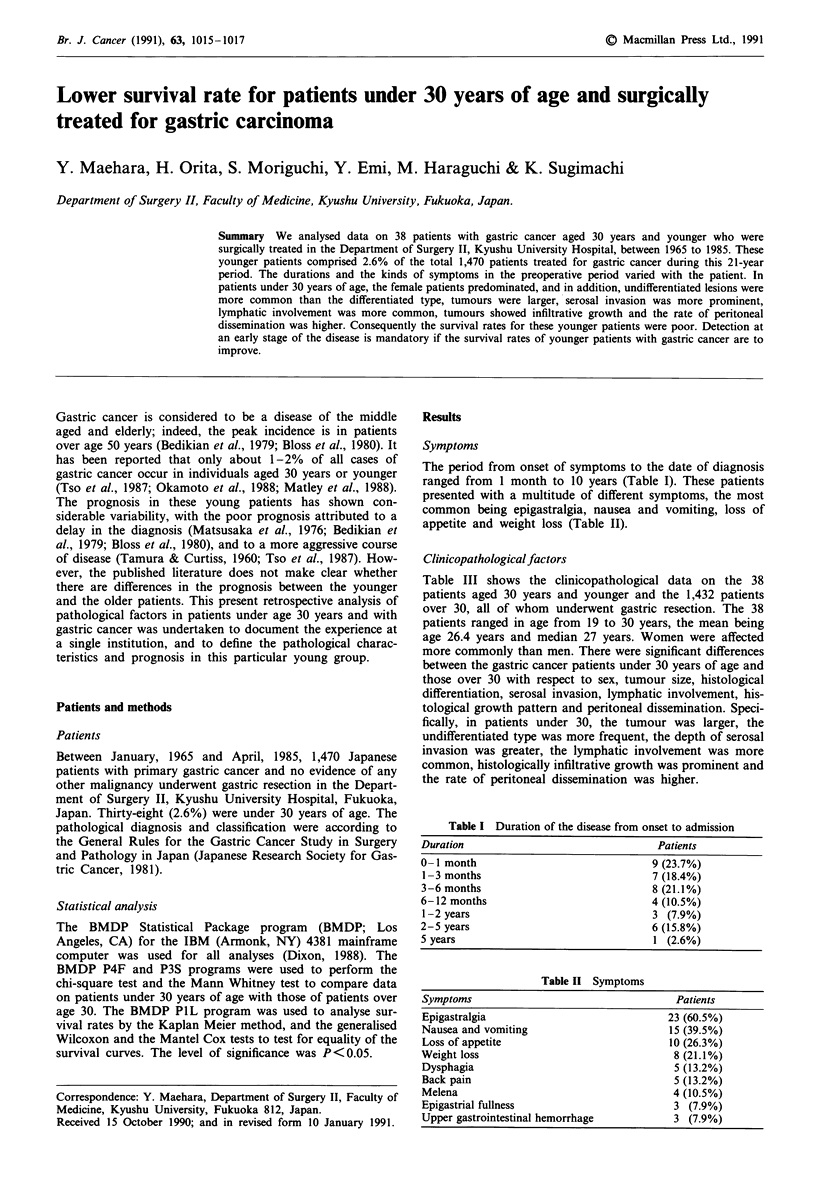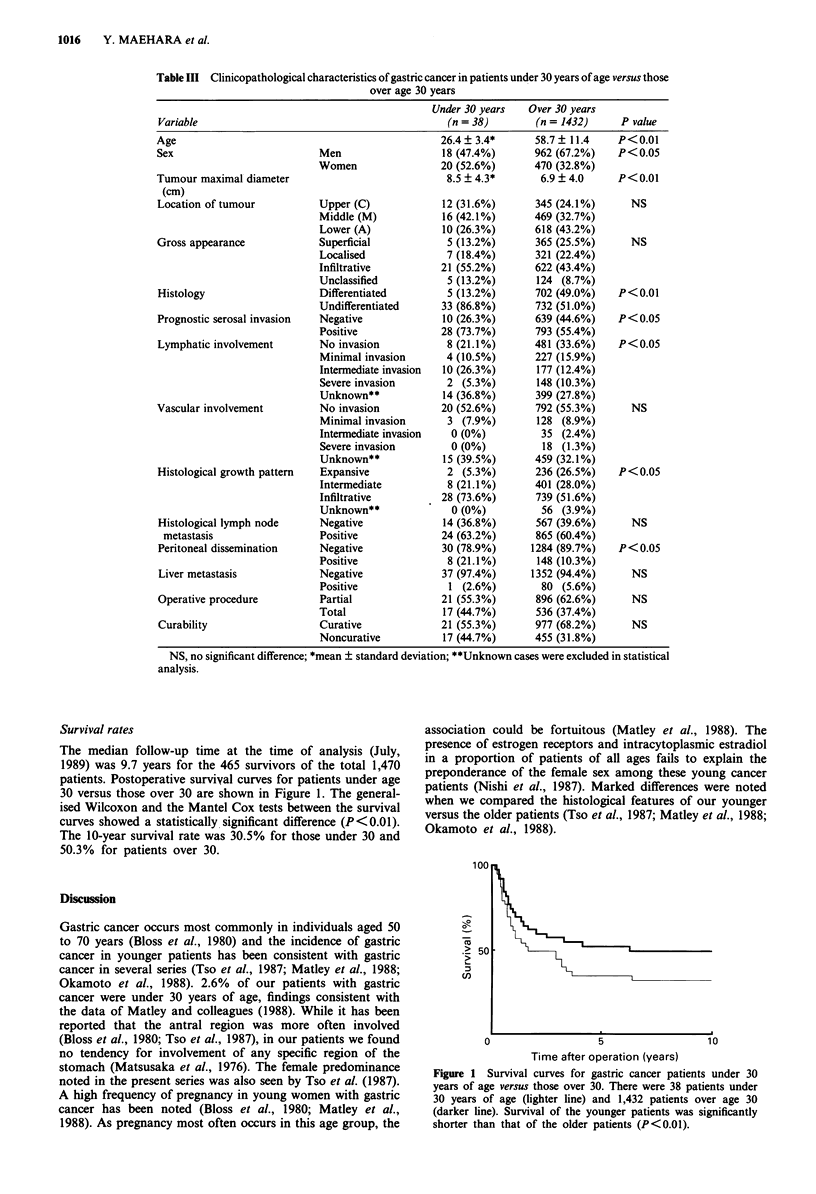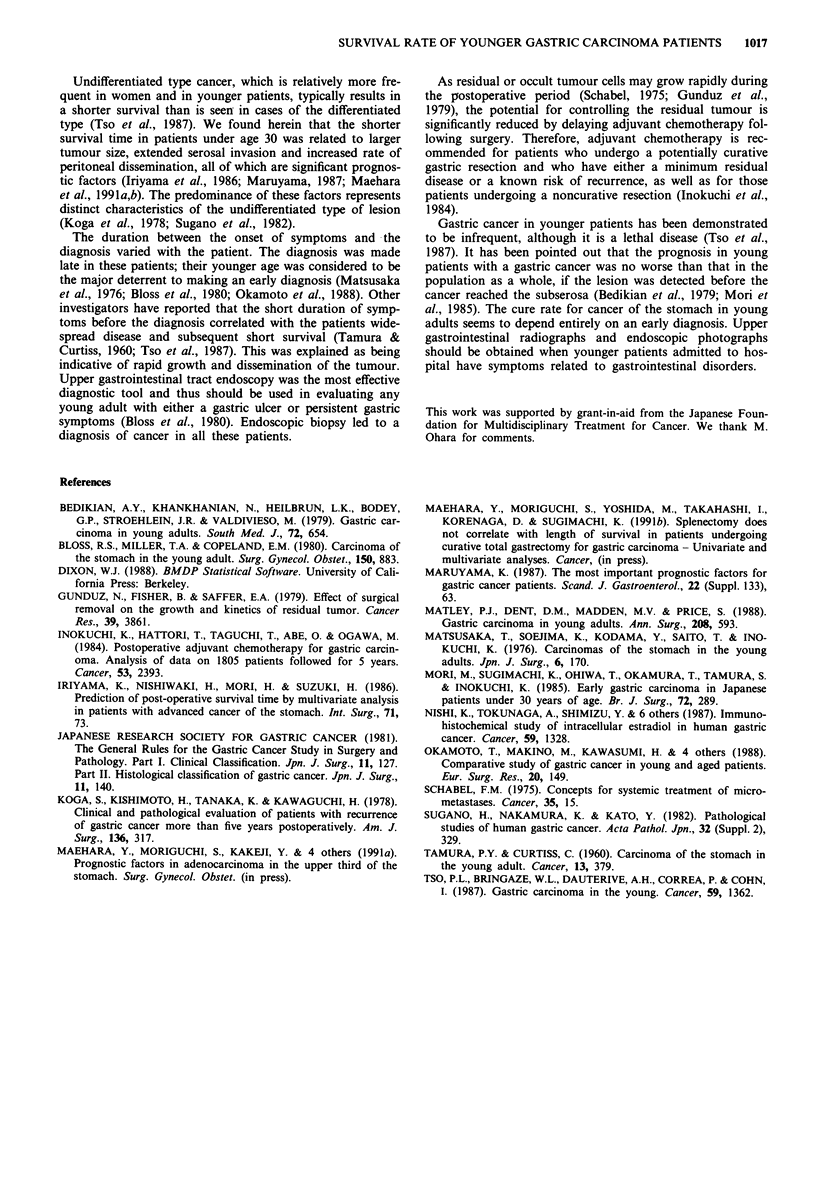# Lower survival rate for patients under 30 years of age and surgically treated for gastric carcinoma.

**DOI:** 10.1038/bjc.1991.220

**Published:** 1991-06

**Authors:** Y. Maehara, H. Orita, S. Moriguchi, Y. Emi, M. Haraguchi, K. Sugimachi

**Affiliations:** Department of Surgery II, Faculty of Medicine, Kyushu University, Fukuoka, Japan.

## Abstract

We analysed data on 38 patients with gastric cancer aged 30 years and younger who were surgically treated in the Department of Surgery II, Kyushu University Hospital, between 1965 to 1985. These younger patients comprised 2.6% of the total 1,470 patients treated for gastric cancer during this 21-year period. The durations and the kinds of symptoms in the preoperative period varied with the patient. In patients under 30 years of age, the female patients predominated, and in addition, undifferentiated lesions were more common than the differentiated type, tumours were larger, serosal invasion was more prominent, lymphatic involvement was more common, tumours showed infiltrative growth and the rate of peritoneal dissemination was higher. Consequently the survival rates for these younger patients were poor. Detection at an early stage of the disease is mandatory if the survival rates of younger patients with gastric cancer are to improve.


Br. J. Cancer (1991), 63, 1015–1017 © Macmillan Press Ltd., 1991

### Lower survival rate for patients under 30 years of age and surgically treated for gastric carcinoma

Y. Maehara, H. Orita, S. Moriguchi, Y. Emi, M. Haraguchi & K. Sugimachi

*Department of Surgery II, Faculty of Medicine, Kyushu University, Fukuoka, Japan.*

**Summary** We analysed data on 38 patients with gastric cancer aged 30 years and younger who were surgically treated in the Department of Surgery II, Kyushu University Hospital, between 1965 to 1985. These younger patients comprised 2.6% of the total 1,470 patients treated for gastric cancer during this 21-year period. The durations and the kinds of symptoms in the preoperative period varied with the patient. In patients under 30 years of age, the female patients predominated, and in addition, undifferentiated lesions were more common than the differentiated type, tumours were larger, serosal invasion was more prominent, lymphatic involvement was more common, tumours showed infiltrative growth and the rate of peritoneal dissemination was higher. Consequently the survival rates for these younger patients were poor. Detection at an early stage of the disease is mandatory if the survival rates of younger patients with gastric cancer are to improve.

Gastric cancer is considered to be a disease of the middle aged and elderly; indeed, the peak incidence is in patients over age 50 years (Bedikian *et al.*, 1979; Bloss *et al.*, 1980). It has been reported that only about 1–2% of all cases of gastric cancer occur in individuals aged 30 years or younger (Tso *et al.*, 1987; Okamoto *et al.*, 1988; Matley *et al.*, 1988). The prognosis in these young patients has shown considerable variability, with the poor prognosis attributed to a delay in the diagnosis (Matsusaka *et al.*, 1976; Bedikian *et al.*, 1979; Bloss *et al.*, 1980), and to a more aggressive course of disease (Tamura & Curtiss, 1960; Tso *et al.*, 1987). However, the published literature does not make clear whether there are differences in the prognosis between the younger and the older patients. This present retrospective analysis of pathological factors in patients under age 30 years and with gastric cancer was undertaken to document the experience at a single institution, and to define the pathological characteristics and prognosis in this particular young group.

### Patients and methods

### *Patients*

Between January, 1965 and April, 1985, 1,470 Japanese patients with primary gastric cancer and no evidence of any other malignancy underwent gastric resection in the Department of Surgery II, Kyushu University Hospital, Fukuoka, Japan. Thirty-eight (2.6%) were under 30 years of age. The pathological diagnosis and classification were according to the General Rules for the Gastric Cancer Study in Surgery and Pathology in Japan (Japanese Research Society for Gastric Cancer, 1981).

### *Statistical analysis*

The BMDP Statistical Package program (BMDP; Los Angeles, CA) for the IBM (Armonk, NY) 4381 mainframe computer was used for all analyses (Dixon, 1988). The BMDP P4F and P3S programs were used to perform the chi-square test and the Mann Whitney test to compare data on patients under 30 years of age with those of patients over age 30. The BMDP P1L program was used to analyse survival rates by the Kaplan Meier method, and the generalised Wilcoxon and the Mantel Cox tests to test for equality of the survival curves. The level of significance was $P < 0.05$.

Correspondence: Y. Maehara, Department of Surgery II, Faculty of Medicine, Kyushu University, Fukuoka 812, Japan.
Received 15 October 1990; and in revised form 10 January 1991.

### Results

### *Symptoms*

The period from onset of symptoms to the date of diagnosis ranged from 1 month to 10 years (Table I). These patients presented with a multitude of different symptoms, the most common being epigastralgia, nausea and vomiting, loss of appetite and weight loss (Table II).

### *Clinicopathological factors*

Table III shows the clinicopathological data on the 38 patients aged 30 years and younger and the 1,432 patients over 30, all of whom underwent gastric resection. The 38 patients ranged in age from 19 to 30 years, the mean being age 26.4 years and median 27 years. Women were affected more commonly than men. There were significant differences between the gastric cancer patients under 30 years of age and those over 30 with respect to sex, tumour size, histological differentiation, serosal invasion, lymphatic involvement, histological growth pattern and peritoneal dissemination. Specifically, in patients under 30, the tumour was larger, the undifferentiated type was more frequent, the depth of serosal invasion was greater, the lymphatic involvement was more common, histologically infiltrative growth was prominent and the rate of peritoneal dissemination was higher.

**Table I** Duration of the disease from onset to admission

| *Duration* | *Patients* |
|---|---|
| 0–1 month | 9 (23.7%) |
| 1–3 months | 7 (18.4%) |
| 3–6 months | 8 (21.1%) |
| 6–12 months | 4 (10.5%) |
| 1–2 years | 3 (7.9%) |
| 2–5 years | 6 (15.8%) |
| 5 years | 1 (2.6%) |

**Table II** Symptoms

| *Symptoms* | *Patients* |
|---|---|
| Epigastralgia | 23 (60.5%) |
| Nausea and vomiting | 15 (39.5%) |
| Loss of appetite | 10 (26.3%) |
| Weight loss | 8 (21.1%) |
| Dysphagia | 5 (13.2%) |
| Back pain | 5 (13.2%) |
| Melena | 4 (10.5%) |
| Epigastrial fullness | 3 (7.9%) |
| Upper gastrointestinal hemorrhage | 3 (7.9%) |

**Table III** Clinicopathological characteristics of gastric cancer in patients under 30 years of age *versus* those over age 30 years

| *Variable* | | *Under 30 years (n = 38)* | *Over 30 years (n = 1432)* | *P value* |
|---|---|---|---|---|
| Age | | 26.4 ± 3.4* | 58.7 ± 11.4 | $P<0.01$ |
| Sex | Men | 18 (47.4%) | 962 (67.2%) | $P<0.05$ |
| | Women | 20 (52.6%) | 470 (32.8%) | |
| Tumour maximal diameter (cm) | | 8.5 ± 4.3* | 6.9 ± 4.0 | $P<0.01$ |
| Location of tumour | Upper (C) | 12 (31.6%) | 345 (24.1%) | NS |
| | Middle (M) | 16 (42.1%) | 469 (32.7%) | |
| | Lower (A) | 10 (26.3%) | 618 (43.2%) | |
| Gross appearance | Superficial | 5 (13.2%) | 365 (25.5%) | NS |
| | Localised | 7 (18.4%) | 321 (22.4%) | |
| | Infiltrative | 21 (55.2%) | 622 (43.4%) | |
| | Unclassified | 5 (13.2%) | 124 (8.7%) | |
| Histology | Differentiated | 5 (13.2%) | 702 (49.0%) | $P<0.01$ |
| | Undifferentiated | 33 (86.8%) | 732 (51.0%) | |
| Prognostic serosal invasion | Negative | 10 (26.3%) | 639 (44.6%) | $P<0.05$ |
| | Positive | 28 (73.7%) | 793 (55.4%) | |
| Lymphatic involvement | No invasion | 8 (21.1%) | 481 (33.6%) | $P<0.05$ |
| | Minimal invasion | 4 (10.5%) | 227 (15.9%) | |
| | Intermediate invasion | 10 (26.3%) | 177 (12.4%) | |
| | Severe invasion | 2 (5.3%) | 148 (10.3%) | |
| | Unknown** | 14 (36.8%) | 399 (27.8%) | |
| Vascular involvement | No invasion | 20 (52.6%) | 792 (55.3%) | NS |
| | Minimal invasion | 3 (7.9%) | 128 (8.9%) | |
| | Intermediate invasion | 0 (0%) | 35 (2.4%) | |
| | Severe invasion | 0 (0%) | 18 (1.3%) | |
| | Unknown** | 15 (39.5%) | 459 (32.1%) | |
| Histological growth pattern | Expansive | 2 (5.3%) | 236 (26.5%) | $P<0.05$ |
| | Intermediate | 8 (21.1%) | 401 (28.0%) | |
| | Infiltrative | 28 (73.6%) | 739 (51.6%) | |
| | Unknown** | 0 (0%) | 56 (3.9%) | |
| Histological lymph node metastasis | Negative | 14 (36.8%) | 567 (39.6%) | NS |
| | Positive | 24 (63.2%) | 865 (60.4%) | |
| Peritoneal dissemination | Negative | 30 (78.9%) | 1284 (89.7%) | $P<0.05$ |
| | Positive | 8 (21.1%) | 148 (10.3%) | |
| Liver metastasis | Negative | 37 (97.4%) | 1352 (94.4%) | NS |
| | Positive | 1 (2.6%) | 80 (5.6%) | |
| Operative procedure | Partial | 21 (55.3%) | 896 (62.6%) | NS |
| | Total | 17 (44.7%) | 536 (37.4%) | |
| Curability | Curative | 21 (55.3%) | 977 (68.2%) | NS |
| | Noncurative | 17 (44.7%) | 455 (31.8%) | |

NS, no significant difference; *mean ± standard deviation; **Unknown cases were excluded in statistical analysis.

### *Survival rates*

The median follow-up time at the time of analysis (July, 1989) was 9.7 years for the 465 survivors of the total 1,470 patients. Postoperative survival curves for patients under age 30 versus those over 30 are shown in Figure 1. The generalised Wilcoxon and the Mantel Cox tests between the survival curves showed a statistically significant difference ($P<0.01$). The 10-year survival rate was 30.5% for those under 30 and 50.3% for patients over 30.

### Discussion

Gastric cancer occurs most commonly in individuals aged 50 to 70 years (Bloss *et al.*, 1980) and the incidence of gastric cancer in younger patients has been consistent with gastric cancer in several series (Tso *et al.*, 1987; Matley *et al.*, 1988; Okamoto *et al.*, 1988). 2.6% of our patients with gastric cancer were under 30 years of age, findings consistent with the data of Matley and colleagues (1988). While it has been reported that the antral region was more often involved (Bloss *et al.*, 1980; Tso *et al.*, 1987), in our patients we found no tendency for involvement of any specific region of the stomach (Matsusaka *et al.*, 1976). The female predominance noted in the present series was also seen by Tso *et al.* (1987). A high frequency of pregnancy in young women with gastric cancer has been noted (Bloss *et al.*, 1980; Matley *et al.*, 1988). As pregnancy most often occurs in this age group, the association could be fortuitous (Matley *et al.*, 1988). The presence of estrogen receptors and intracytoplasmic estradiol in a proportion of patients of all ages fails to explain the preponderance of the female sex among these young cancer patients (Nishi *et al.*, 1987). Marked differences were noted when we compared the histological features of our younger versus the older patients (Tso *et al.*, 1987; Matley *et al.*, 1988; Okamoto *et al.*, 1988).

**Figure 1** Survival curves for gastric cancer patients under 30 years of age *versus* those over 30. There were 38 patients under 30 years of age (lighter line) and 1,432 patients over age 30 (darker line). Survival of the younger patients was significantly shorter than that of the older patients ($P<0.01$).

Undifferentiated type cancer, which is relatively more frequent in women and in younger patients, typically results in a shorter survival than is seen in cases of the differentiated type (Tso *et al.*, 1987). We found herein that the shorter survival time in patients under age 30 was related to larger tumour size, extended serosal invasion and increased rate of peritoneal dissemination, all of which are significant prognostic factors (Iriyama *et al.*, 1986; Maruyama, 1987; Maehara *et al.*, 1991*a*,*b*). The predominance of these factors represents distinct characteristics of the undifferentiated type of lesion (Koga *et al.*, 1978; Sugano *et al.*, 1982).

The duration between the onset of symptoms and the diagnosis varied with the patient. The diagnosis was made late in these patients; their younger age was considered to be the major deterrent to making an early diagnosis (Matsusaka *et al.*, 1976; Bloss *et al.*, 1980; Okamoto *et al.*, 1988). Other investigators have reported that the short duration of symptoms before the diagnosis correlated with the patients widespread disease and subsequent short survival (Tamura & Curtiss, 1960; Tso *et al.*, 1987). This was explained as being indicative of rapid growth and dissemination of the tumour. Upper gastrointestinal tract endoscopy was the most effective diagnostic tool and thus should be used in evaluating any young adult with either a gastric ulcer or persistent gastric symptoms (Bloss *et al.*, 1980). Endoscopic biopsy led to a diagnosis of cancer in all these patients.

As residual or occult tumour cells may grow rapidly during the postoperative period (Schabel, 1975; Gunduz *et al.*, 1979), the potential for controlling the residual tumour is significantly reduced by delaying adjuvant chemotherapy following surgery. Therefore, adjuvant chemotherapy is recommended for patients who undergo a potentially curative gastric resection and who have either a minimum residual disease or a known risk of recurrence, as well as for those patients undergoing a noncurative resection (Inokuchi *et al.*, 1984).

Gastric cancer in younger patients has been demonstrated to be infrequent, although it is a lethal disease (Tso *et al.*, 1987). It has been pointed out that the prognosis in young patients with a gastric cancer was no worse than that in the population as a whole, if the lesion was detected before the cancer reached the subserosa (Bedikian *et al.*, 1979; Mori *et al.*, 1985). The cure rate for cancer of the stomach in young adults seems to depend entirely on an early diagnosis. Upper gastrointestinal radiographs and endoscopic photographs should be obtained when younger patients admitted to hospital have symptoms related to gastrointestinal disorders.

This work was supported by grant-in-aid from the Japanese Foundation for Multidisciplinary Treatment for Cancer. We thank M. Ohara for comments.

### References

BEDIKIAN, A.Y., KHANKHANIAN, N., HEILBRUN, L.K., BODEY, G.P., STROEHLEIN, J.R. & VALDIVIESO, M. (1979). Gastric carcinoma in young adults. *South Med. J.*, **72**, 654.

BLOSS, R.S., MILLER, T.A. & COPELAND, E.M. (1980). Carcinoma of the stomach in the young adult. *Surg. Gynecol. Obstet.*, **150**, 883.

DIXON, W.J. (1988). *BMDP Statistical Software*. University of California Press: Berkeley.

GUNDUZ, N., FISHER, B. & SAFFER, E.A. (1979). Effect of surgical removal on the growth and kinetics of residual tumor. *Cancer Res.*, **39**, 3861.

INOKUCHI, K., HATTORI, T., TAGUCHI, T., ABE, O. & OGAWA, M. (1984). Postoperative adjuvant chemotherapy for gastric carcinoma. Analysis of data on 1805 patients followed for 5 years. *Cancer*, **53**, 2393.

IRIYAMA, K., NISHIWAKI, H., MORI, H. & SUZUKI, H. (1986). Prediction of post-operative survival time by multivariate analysis in patients with advanced cancer of the stomach. *Int. Surg.*, **71**, 73.

JAPANESE RESEARCH SOCIETY FOR GASTRIC CANCER (1981). The General Rules for the Gastric Cancer Study in Surgery and Pathology. Part I. Clinical Classification. *Jpn. J. Surg.*, **11**, 127. Part II. Histological classification of gastric cancer. *Jpn. J. Surg.*, **11**, 140.

KOGA, S., KISHIMOTO, H., TANAKA, K. & KAWAGUCHI, H. (1978). Clinical and pathological evaluation of patients with recurrence of gastric cancer more than five years postoperatively. *Am. J. Surg.*, **136**, 317.

MAEHARA, Y., MORIGUCHI, S., KAKEJI, Y. & 4 others (1991*a*). Prognostic factors in adenocarcinoma in the upper third of the stomach. *Surg. Gynecol. Obstet.* (in press).

MAEHARA, Y., MORIGUCHI, S., YOSHIDA, M., TAKAHASHI, I., KORENAGA, D. & SUGIMACHI, K. (1991*b*). Splenectomy does not correlate with length of survival in patients undergoing curative total gastrectomy for gastric carcinoma – Univariate and multivariate analyses. *Cancer*, (in press).

MARUYAMA, K. (1987). The most important prognostic factors for gastric cancer patients. *Scand. J. Gastroenterol.*, **22** (Suppl. 133), 63.

MATLEY, P.J., DENT, D.M., MADDEN, M.V. & PRICE, S. (1988). Gastric carcinoma in young adults. *Ann. Surg.*, **208**, 593.

MATSUSAKA, T., SOEJIMA, K., KODAMA, Y., SAITO, T. & INOKUCHI, K. (1976). Carcinomas of the stomach in the young adults. *Jpn. J. Surg.*, **6**, 170.

MORI, M., SUGIMACHI, K., OHIWA, T., OKAMURA, T., TAMURA, S. & INOKUCHI, K. (1985). Early gastric carcinoma in Japanese patients under 30 years of age. *Br. J. Surg.*, **72**, 289.

NISHI, K., TOKUNAGA, A., SHIMIZU, Y. & 6 others (1987). Immunohistochemical study of intracellular estradiol in human gastric cancer. *Cancer*, **59**, 1328.

OKAMOTO, T., MAKINO, M., KAWASUMI, H. & 4 others (1988). Comparative study of gastric cancer in young and aged patients. *Eur. Surg. Res.*, **20**, 149.

SCHABEL, F.M. (1975). Concepts for systemic treatment of micrometastases. *Cancer*, **35**, 15.

SUGANO, H., NAKAMURA, K. & KATO, Y. (1982). Pathological studies of human gastric cancer. *Acta Pathol. Jpn.*, **32** (Suppl. 2), 329.

TAMURA, P.Y. & CURTISS, C. (1960). Carcinoma of the stomach in the young adult. *Cancer*, **13**, 379.

TSO, P.L., BRINGAZE, W.L., DAUTERIVE, A.H., CORREA, P. & COHN, I. (1987). Gastric carcinoma in the young. *Cancer*, **59**, 1362.